# Highly Sensitive Control Study of PD Archimedean Antenna Based on Rotating Unit Reflective Metasurface

**DOI:** 10.3390/mi17030363

**Published:** 2026-03-17

**Authors:** Lihao Luo, Junlin Gai, Dapeng Han, Minghan Ke, Haonan Zhang, Zhenhao Huang, Guozhi Zhang

**Affiliations:** 1 Hubei Provincial Engineering Research Center for New Energy and Grid Equipment Safety Monitoring, Hubei University of Technology, Wuhan 430068, China; 18102336261@163.com (L.L.); 18902296232@139.com (J.G.); 17622335969@163.com (D.H.); 13387560942@163.com (M.K.); 13035139268@163.com (H.Z.); 13018053772@163.com (Z.H.); 2 Hubei Provincial Key Laboratory of Solar Energy Efficient Utilization and Energy Storage Operation Control, Hubei University of Technology, Wuhan 430068, China

**Keywords:** partial discharge, Archimedean antenna, metasurface control, gain, discharge detection

## Abstract

Addressing the insufficient sensitivity of typical Archimedean spiral antennas for detecting partial discharge (PD) in electrical equipment, this paper proposes a high-sensitivity regulation technique for PD Archimedean antennas based on rotating unit-cell reflective metasurfaces. First, a finite element model of the ultra-high-frequency Archimedean antenna was constructed. Then, employing metasurface electromagnetic wave reflection technology and phase compensation principles, a rotating-unit reflective metasurface was designed to optimize its full-bandwidth gain. A multi-parameter joint optimization method was used to obtain the optimal data for the antenna and metasurface parameters. Finally, simulations and experimental analyses of the super-surface-controlled Archimedean antenna revealed the following: The gain of the Archimedean antenna controlled by the rotating-unit super-surface increases by up to 15.61 dB in the 0.3–1.5 GHz band, with an average full-band gain enhancement of 3.42 dB. During electrostatic discharge (ESD), the amplitude of UHF signals detected by the Archimedean antenna increases by approximately 88.9%, and the amplitude detection of UHF signals during GIS discharges increases by approximately 138.6–150%. These results demonstrate that the metasurface significantly enhances the antenna’s gain performance, providing a reference for highly sensitive control technologies in detecting discharges in electrical equipment.

## 1. Introduction

Substations serve as the core hubs of power systems, acting as critical nodes for electricity transmission and distribution in modern grids. However, due to influences from equipment manufacturing, installation, operation, maintenance, and environmental conditions, power equipment inevitably develops insulation defects. Partial discharge (PD) occurs when insulation defects disrupt the uniform electric field on or within the insulating medium, leading to localized atmospheric ionization or dielectric breakdown. This discharge continuously damages power equipment in a concealed manner [1,2,3,4].

Online monitoring of PD in electrical equipment is a critical aspect of substation operation and maintenance. PD detection methods are primarily categorized into electrical and non-electrical approaches [4,5,6]. Electrical methods include ultra-high frequency (UHF), very high frequency (VHF), impulse current (IEC 60270), and transient earth wave (TEW) techniques. Non-electrical methods encompass acoustic (ultrasonic), optical, and infrared detection. Among these, the UHF method detects electromagnetic pulse signals primarily within the 0.3–1.5 GHz frequency band [7], effectively avoiding electromagnetic interference from corona discharges at the site. It offers advantages such as strong anti-interference capability and high reliability, leading to its widespread application in power sites. However, it also has insufficient sensing sensitivity for UHF antenna sensors.

To enhance the detection sensitivity of UHF antenna sensors, numerous studies have been conducted worldwide: Muhammad Wasif Niaz from Pakistan proposed using a resonant cavity to convert the antenna’s omnidirectional or wide-angle radiation into directional radiation, achieving spatial energy focusing and ultimately gaining enhancement at the resonant frequency [8]. However, this approach suffers from narrow bandwidth, an upper limit on gain enhancement, and rapidly diminishing marginal benefits. Ma Shijin from Sichuan University proposed adding a dielectric coating to the antenna. By leveraging the dielectric properties of the material, this method modulates the antenna’s radiation wavefront, optimizes current distribution, suppresses surface wave losses, and enhances the field strength superposition of directional radiation, thereby increasing antenna gain [9]. However, this approach demands stringent control over dielectric parameters and installation precision and faces limitations in achieving significant gain enhancement at high frequencies. Researchers, including C. Shen from Beijing University of Posts and Telecommunications, C. Wang from Tianjin University, A. K. Ojha from India, and Q. Wu from Shenzhen University, employed resonators to enable broadband gain jumps [10,11,12,13]. Yet the inherently narrow-band nature of resonators conflicts with the broad UHF requirements. Researchers, including G. Peeler (USA), E. Erfani (Canada), and C. Lee (South Korea), proposed lens-based approaches to convert the spherical waves radiated by feed sources into plane waves, thereby compressing beam width, enhancing energy concentration and directivity, and ultimately increasing antenna gain [14,15,16]. Compared to resonant cavities and resonators, this lens approach offers significant advantages in bandwidth, loss, and beam flexibility. However, core limitations persist, including size-gain coupling, increased dielectric loss with frequency, limited beam scanning adaptability, high manufacturing precision requirements, and wide-angle incidence distortion.

Based on this, this paper proposes a high-sensitivity control technique for PD UHF antennas utilizing rotating-unit reflective metasurfaces [17,18,19]. First, a finite element model of the UHF Archimedean spiral antenna is constructed. Then, employing metasurface electromagnetic wave reflection technology and phase compensation principles, a rotating-unit reflective metasurface is designed to optimize its full-bandwidth gain. A multi-parameter joint optimization method is adopted to obtain optimal data for the antenna and metasurface parameters. Finally, a prototype of the metasurface-controlled PD UHF Archimedean helical antenna is fabricated and tested on a full-scale GIS PD simulation platform for evaluation and analysis.

## 2. PD Archimedean Helical Antenna and Modeling Simulation

The core structure of the Archimedean helical antenna consists of two or more metal arms developed according to the Archimedean spiral equations. The equations for two Archimedean spiral arms are given by Equations (1) and (2):

(1) $$r=r_{0}+a\theta$$

where *r* is the distance from a point on the helix to the origin; *θ* is the polar angle (in radians); *r*_0_ is the initial radius (helix starting position); and *a* is the helix growth rate (determining the helix pitch).

The other arm is obtained by rotating the first arm 180 degrees. The equation form is as follows:

(2) $$r=r_{0}+a\left(\theta -\pi \right)$$

The operating frequency band of an Archimedean antenna is determined by its dimensions. Its low-frequency cutoff *λ_L_* depends on the maximum radius *r_(M)_* of the spiral arms, typically given by the following:

(3) $$2\pi r_{M}\geq 1.25\lambda_{L}$$

The high-frequency cutoff *λ_H_* depends on the inner radius *r*_0_ of the spiral arms, typically given by the following:

(4) $$2r_{0}\leq \lambda_{H}/4$$

The primary frequency energy spectrum of PD-radiated electromagnetic pulses is concentrated between 0.3 and 1.5 GHz. Equation (3) indicates *r_M_* is approximately 200 mm. However, cost, space constraints, weight, aerodynamic drag, or specific application requirements necessitate minimizing antenna dimensions. Therefore, this paper designs the Archimedean antenna with a starting frequency of fl = 0.6 GHz. According to Equation (3), the antenna’s design starting frequency of 0.6 GHz corresponds to a maximum diameter of 2*r_M_* = 200 mm. To further reduce the antenna radius, the spiral arms are bent starting from the 6th turn [20], ending at the 12th turn. This achieves a maximum antenna radius of 77 mm, realizing the miniaturization of the Archimedean antenna. *r*_0_ was set to 1/2 *λ_H_*/4, i.e., 12.5 mm. The spiral arm width w and spacing s were set to 1.5 mm, forming a self-complementary structure. The dielectric substrate thickness d was selected as the standard 1.6 mm.

**Figure 1:**
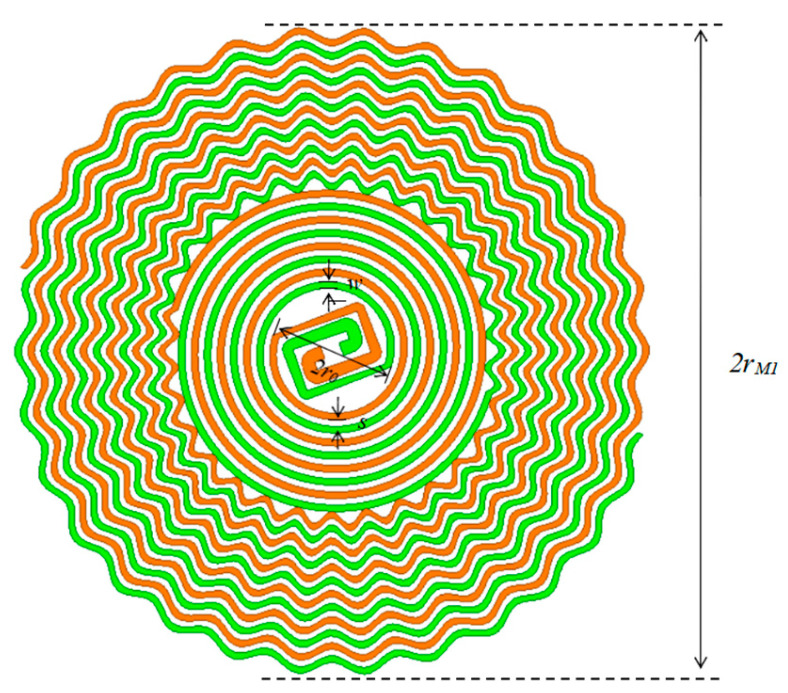
Coplanar microstrip gradually varying balun sinusoidal wave corrugated Archimedean antenna.

Due to the large spacing between the starting points of the two helical arms, two microstrip lines are used for feeding, as shown in Figure 1. Modeling was completed in HFSS. The scaled-down helical arm model and antenna parameters are shown in Figure 1 and Table 1, respectively.

**Figure 2:**
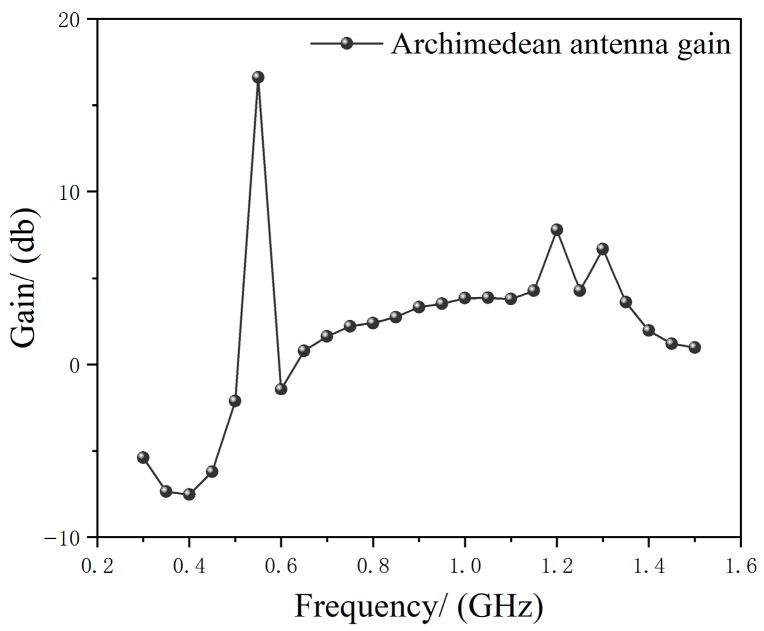
Archimedean Antenna Simulation Gain in the 0.3–1.5 GHz Frequency Band.

After modeling completion, simulation analysis was conducted on the Archimedean antenna. The maximum gain characteristics for each direction within the 0.3–1.5 GHz frequency band are shown in Figure 2.

Figure 2 shows the maximum gain of the Archimedean antenna in all directions across the 0.3–1.5 GHz frequency band. It can be observed that the gain is relatively low in the lower frequency range (<0 dB).

## 3. Rotating Unit Reflective Metasurface Control

### 3.1. Principle of Reflective Metasurface Gain Enhancement

Reflective metasurfaces adhere to generalized Snell’s law and phase modulation mechanisms. The rotating unit structure is designed based on phase compensation principles to achieve phase modulation, thereby introducing spatial phase gradients into the metasurface. This overcomes the constraint of the traditional reflection law, where the angle of incidence equals the angle of reflection, causing the reflected wave to undergo anomalous deflection as described by Equation (5).

(5) $$\sin\theta_{r}-\sin\theta_{i}=\frac{\lambda_{0}}{2\pi }\frac{dp}{dx}$$

**Figure 3:**
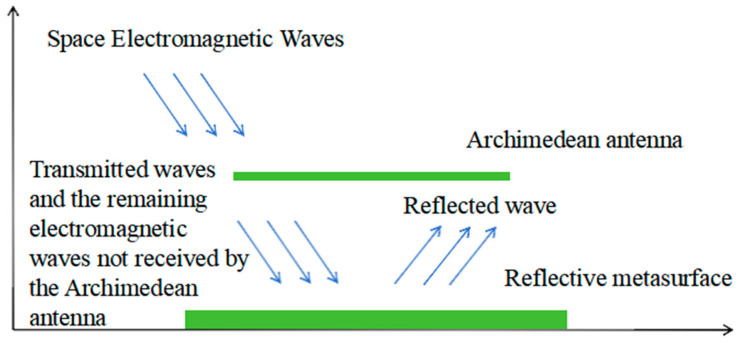
Principle of gain enhancement through reflective Metasurfaces.

Based on the above principle of the reflective metasurface, a portion of the electromagnetic waves in space is directly received by the Archimedean antenna, while another portion propagates backward through the Archimedean antenna. Simultaneously, a significant number of electromagnetic waves in space are neither received nor transmitted by the Archimedean antenna. The reflective metasurface enhances the gain of the Archimedean antenna by reflecting these two portions of electromagnetic waves toward it, as shown in Figure 3.

### 3.2. Design of Rotating Unit Reflective Metasurfaces

**Figure 4:**
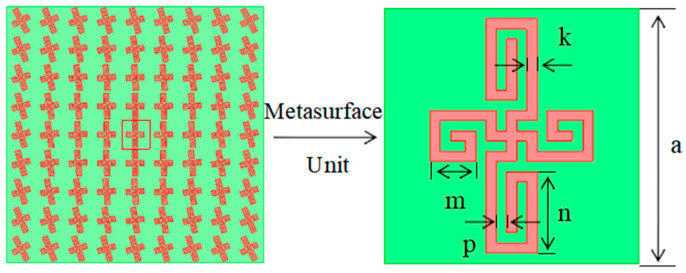
Metasurface structure diagram.

Based on phase compensation principles, a single-layer broadband deformable cross-unit was designed to modulate electromagnetic wave phase, as shown in Figure 4 [21]. Considering the need to match the Metasurface dimensions to the Archimedean antenna size, the reflective Metasurface employs unit dimensions of a = 20 mm, deformation cross-arm width (k) and spacing *p* set to 0.8 mm, an x-direction metal branch length of m = 3.6 mm, y-direction metal branch length of *n* = 6.4 mm, metamaterial substrate thickness of d1 = 3 mm, and relative permittivity of 3. The metasurface elements were then rotated to achieve phase compensation. According to the phase compensation principle, the phase distribution that each element should satisfy in the xoy plane is given by Equation (6).

(6) $$\varphi \left(x,y\right)=\frac{2\pi }{\lambda_{0}}\left(\sqrt{x^{2}+y^{2}+f^{2}}-f\right)+\varphi_{0}$$

Therefore, with the metasurface center set as phase 0, the phase compensation at each position on the metasurface is calculated to determine the rotation angle for each unit. The metasurface structure is designed accordingly [21] to correct phase distortion introduced during signal transmission, acquisition, or processing. This restores the true phase characteristics of the signal, enhancing the identification accuracy, localization precision, and gain stability of partial discharge signals. The backside of the metasurface is coated with metallic copper to maintain the amplitude of reflected waves unchanged [21], focusing the energy beam toward the antenna’s main lobe direction to enhance gain. This transforms the bidirectional radiation characteristics of the Archimedean spiral antenna into unidirectional radiation. The metasurface structure and parameters are shown in Figure 4 and Table 2, respectively.

### 3.3. Rotating Unit Reflective Metasurface-Controlled Archimedean Antenna Parameter Optimization

**Figure 5:**
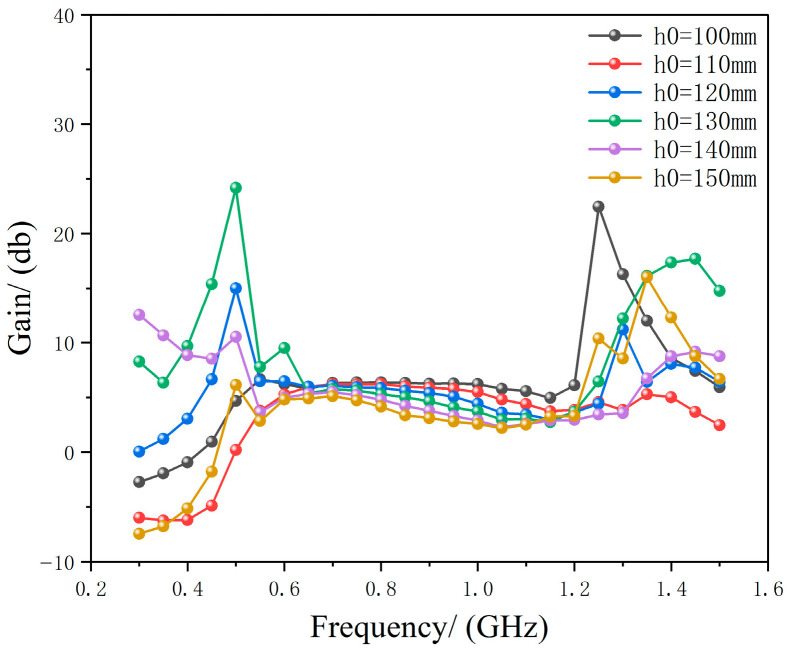
Simulation results of Archimedean antenna gain varying with *h*_0_.

Subsequently, a multi-scale joint optimization method was employed. First, HFSS was used to optimize the distance *h*_0_ between the metasurface and the Archimedean antenna when no physical connection existed between them, obtaining the optimal *h*_0_ value for maximum gain enhancement of the Archimedean antenna by the metasurface. The optimized *h*_0_ result is shown in Figure 5.

As shown in Figure 5, the metasurface provides the best gain enhancement for the Archimedean antenna at *h*_0_ = 130 mm.

In practical applications, the metasurface must be physically connected to the antenna for support. Ideally, the connecting material should have no effect on the gain after covering the metasurface. However, materials that are completely non-absorbing, non-reflective, and electrically insulating do not exist. Therefore, we can only strive to minimize the impact of the support material on performance.

Foam materials like polyethylene foam and polystyrene foam are commonly used as supports. They exhibit weak surface reflection and minimal internal absorption of electromagnetic waves, making them suitable for applications such as antenna supports and radar domes. However, since the actual starting operating frequency of the antenna in this paper is 0.3 GHz—lower than the design starting frequency of 0.6 GHz—the wavelength at 0.3 GHz is 1000 mm. Based on the relationship between the maximum outer radius of the Archimedean antenna’s spiral arms and wavelength, the theoretical outer radius for an antenna operating at 0.3 GHz without spiral arm bending should be 400 mm. Therefore, theoretically, the antenna extends outward to capture electromagnetic waves in the approximate range of 0.3 GHz to 0.6 GHz. Consequently, traditional support methods like foam support would significantly impact antenna performance, a finding validated through HFSS simulation.

**Figure 6:**
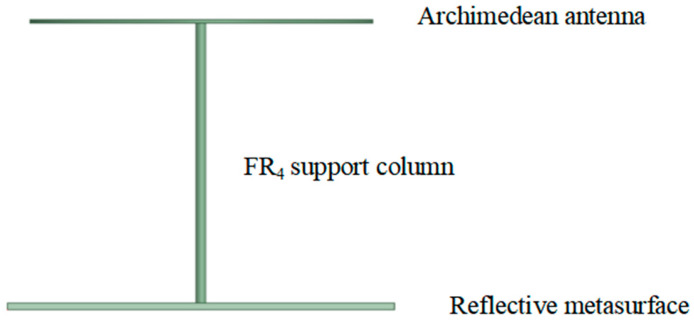
Schematic of the Archimedean antenna structure regulated by the metasurface.

**Figure 7:**
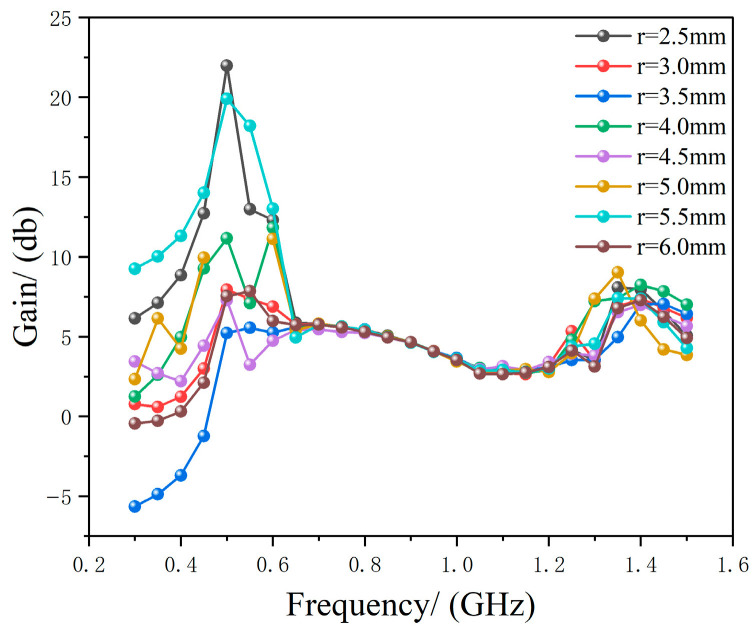
Simulation results of Archimedean antenna gain enhancement with respect to FR_4_ support rod radius.

The antenna has a high-frequency cutoff at 1.5 GHz. The starting radius adopted in this paper is 12.5 mm. Therefore, the central pillar made of FR_4_ material is used to support both the antenna and the pillar. Since the phase at the geometric center of the metasurface is zero, this metasurface element is removed to connect the pillar. While maintaining the fixed parameters of and *h*_0_ = 130 mm, the pillar radius *r_1_* is optimized via HFSS to minimize the impact on the Archimedean antenna performance. The support structure and optimization results are shown in Figure 6 and Figure 7, respectively.

**Figure 8:**
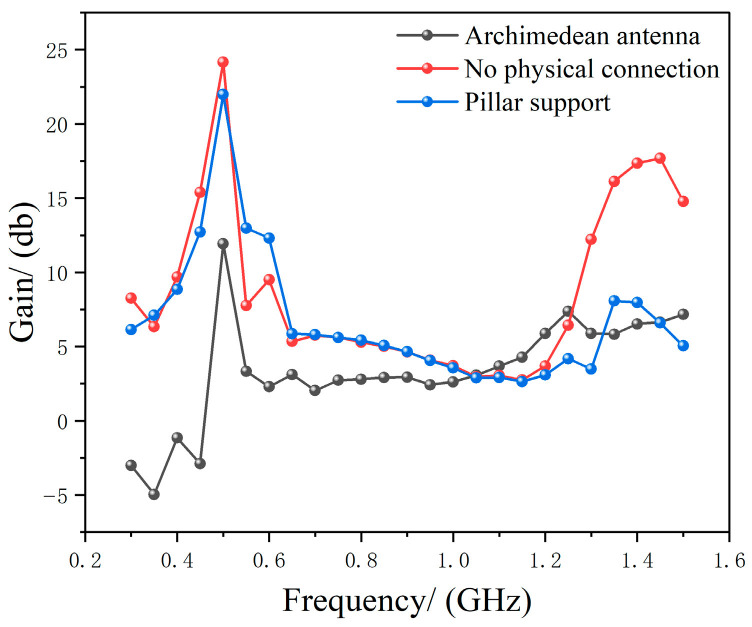
Final gain comparison.

As shown in Figure 7, when *r*_1_ = 2.5 mm, the FR_4_ material support rods exert a negligible influence on antenna gain. Figure 8 compares the gains of three configurations across the 0.3–1.5 GHz frequency band: the Archimedean antenna alone, the Archimedean antenna with metasurface regulation but no physical connection, and the Archimedean antenna with metasurface regulation and 2.5 mm FR_4_ support rods.

As shown in Figure 8, since the strut dimensions are close to the physical size corresponding to the high-frequency cutoff of 1.5 GHz, adding support at high frequencies results in a significant reduction in gain compared to the physically unconnected case. However, at mid-to-low frequencies, where the strut dimensions are far from the physical size corresponding to the frequency, the gain shows no significant change. The optimal configuration was determined to be *h*_0_ = 130 mm and *r*_1_ = 2.5 mm. After adding the metasurface and connecting the supports, the gain was significantly optimized compared to the traditional Archimedean spiral antenna, with a maximum improvement of 15.61 dB. Based on the gain values at each frequency point along the gain-frequency curves before and after metasurface-based tuning, the average gain in the 0.3–1.5 GHz band after tuning increased by approximately 3.42 dB compared to before tuning.

**Figure 9:**
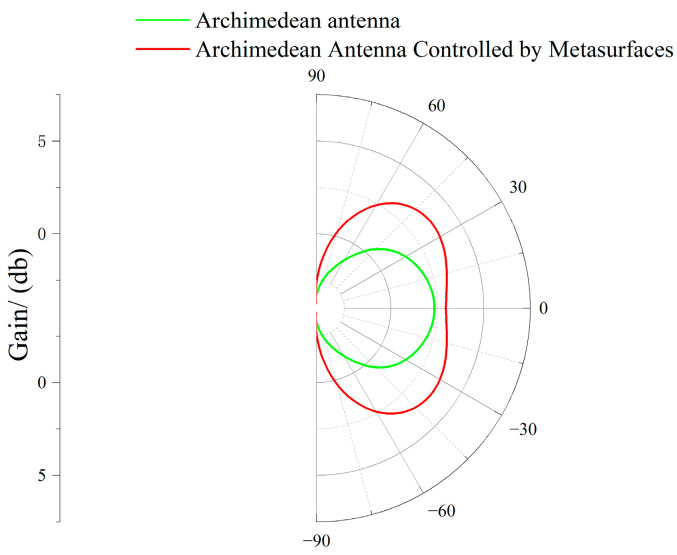
Radiation pattern before and after metasurface control at 1 GHz.

Since the subsequent section employs an oscilloscope to test the enhancement effect of the metasurface on the amplitude detection of UHF signals by the Archimedean antenna at a frequency of 1 GHz, a comparison diagram of the radiation pattern before and after metasurface control at 1 GHz was simulated in HFSS, as shown in Figure 9.

**Figure 10:**
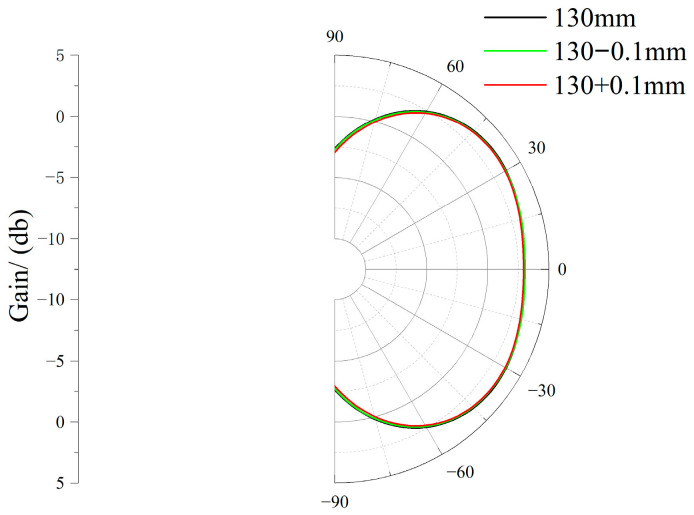
*h*_0_ Tolerance Sensitivity Analysis Radiation Chart.

**Figure 11:**
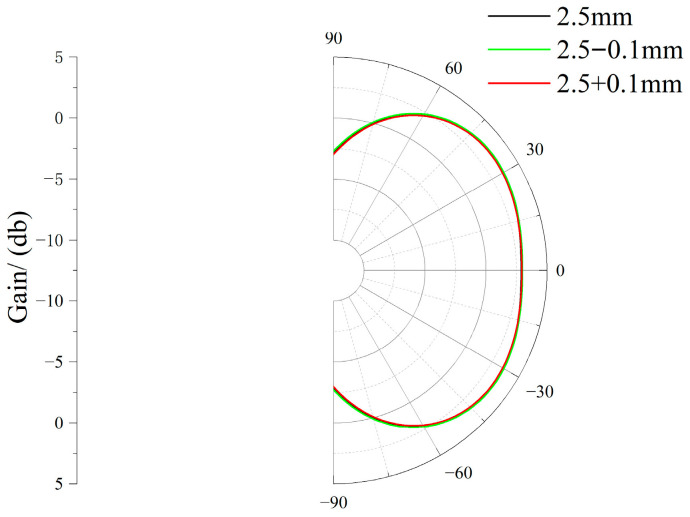
*r*_1_ Tolerance Sensitivity Analysis Radiation Chart.

**Figure 12:**
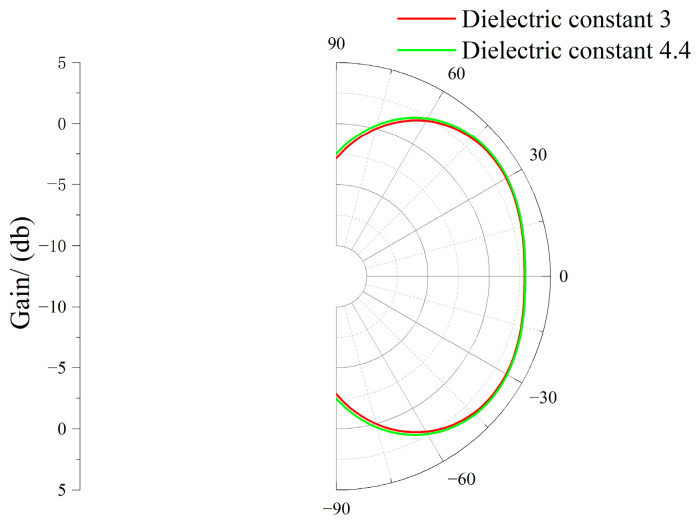
Material Parameter Variation Sensitivity Analysis Radiation Diagram.

To further enhance the reliability of results, sensitivity analysis was conducted on manufacturing tolerances and material parameter variations. The error range in physical production is 0.07 mm ± 0.02 mm, meaning that the error is less than 0.1 mm. Therefore, for the two parameters *h*_0_ and *r*_1_ optimized above that significantly impact performance, the radiation patterns at the 1 GHz frequency band were simulated at ±0.01 mm from the optimal values. Regarding material variation, the dielectric constant of the metasurface dielectric substrate—a parameter significantly affecting performance—was primarily considered. Radiation patterns at the 1 GHz frequency band were simulated and verified for dielectric constants of 3 and 4.4. The radiation patterns for sensitivity analysis of *h*_0_ tolerance, *r*_1_ tolerance, and material parameter variation are shown in Figure 10, Figure 11, and Figure 12, respectively.

As shown in Figure 10, Figure 11 and Figure 12, slight discrepancies between simulation results and physical testing may arise due to manufacturing tolerances and material parameter variations. However, these differences are relatively minor and fall within an acceptable range.

### 3.4. Loss Analysis

**Figure 13:**
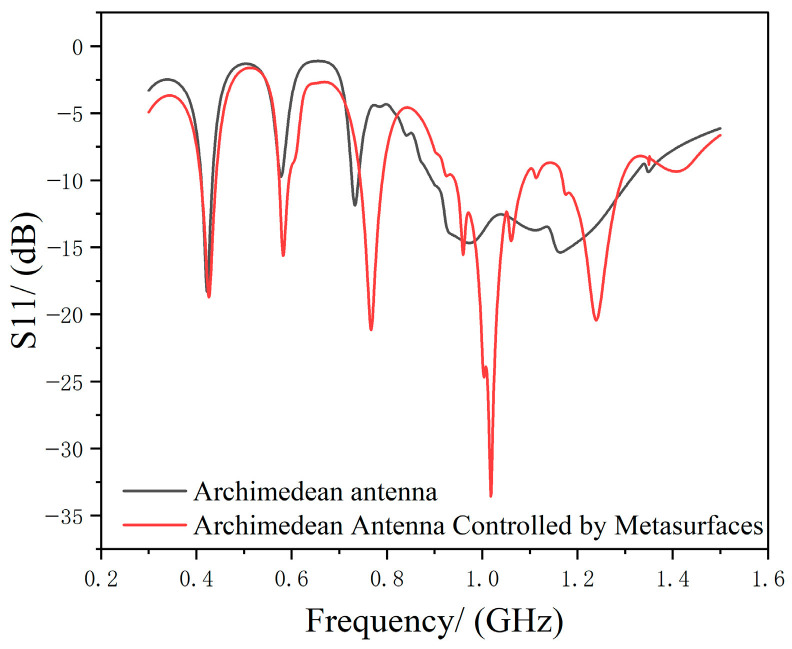
Comparison of Archimedean Antenna Return Loss Before and After Metasurface Modulation.

Although metasurfaces significantly enhance antenna performance, they may also introduce additional losses and potential parasitic radiation. Therefore, we simulated the return loss before and after metasurface modulation, as shown in Figure 13.

As shown in Figure 13, since the metasurface does not alter the antenna’s original structure but merely enhances its gain through an additional attachment, its core impedance matching remains unchanged. Before and after metasurface regulation, the positive and negative fluctuations in the Archimedean Antenna’s return loss remain within normal ranges. Therefore, it can be approximated that the metasurface introduces no additional loss or significant potential parasitic radiation. Future improvements could be achieved by adopting multilayer structures or selecting more suitable materials.

### 3.5. Physical Testing

**Figure 14:**
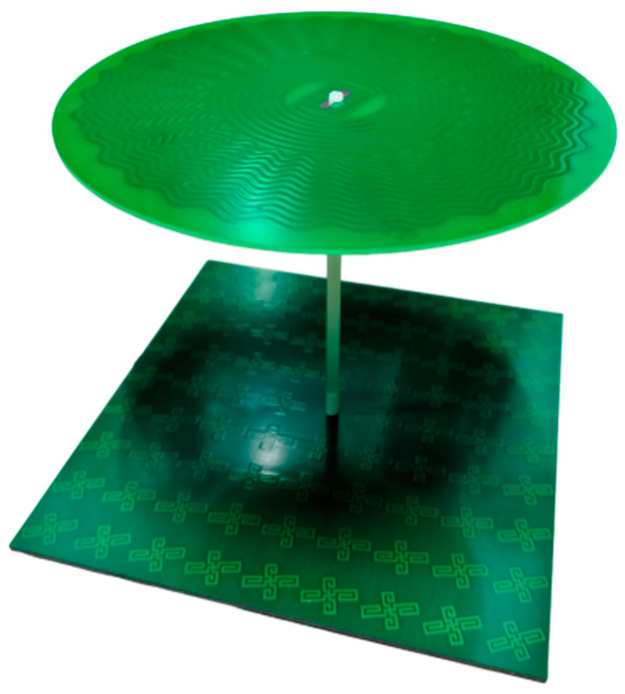
Schematic diagram of the physical structure of the Archimedean antenna controlled by the super surface.

The physical Archimedean antenna with metasurface control is shown in Figure 14. The upper layer is the traditional Archimedean antenna; the lower layer is the rotating unit reflection metasurface; and the middle layer uses FR_4_ pillars for support and connection.

**Figure 15:**
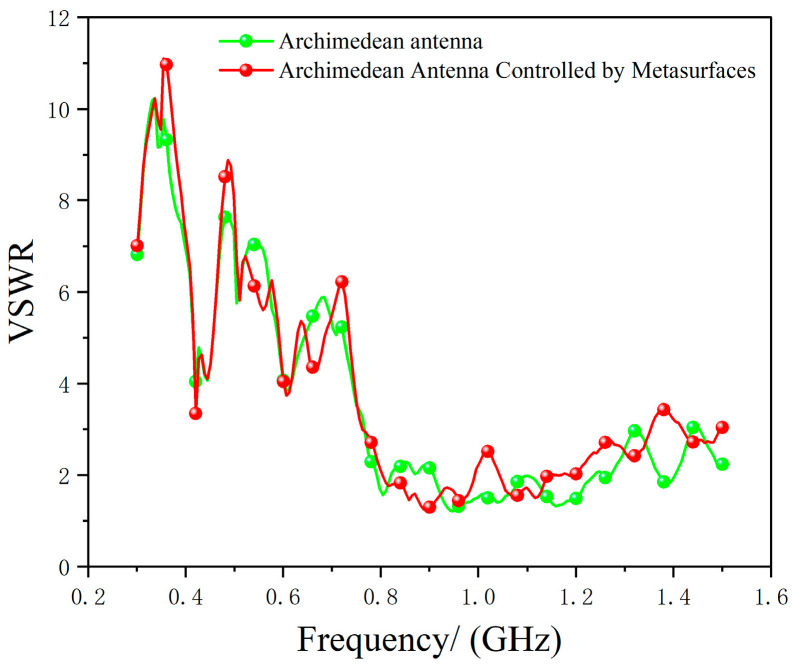
Measured standing wave ratio of Archimedean antenna before and after metasurface modulation.

We first tested the actual return loss, i.e., the standing wave ratio (SWR), of the Archimedean antenna after metasurface modulation. The conversion method is as per Equation (7), and the test results are shown in Figure 15.

(7) $$RL=20\log_{10}(\frac{VSWR+1}{VSWR-1})$$

As shown in Figure 15, the standing wave ratio (VSWR) of the Archimedean antenna remains within normal fluctuation ranges before and after metasurface modulation, verifying the simulation results that the metasurface introduces negligible additional loss.

**Figure 16:**
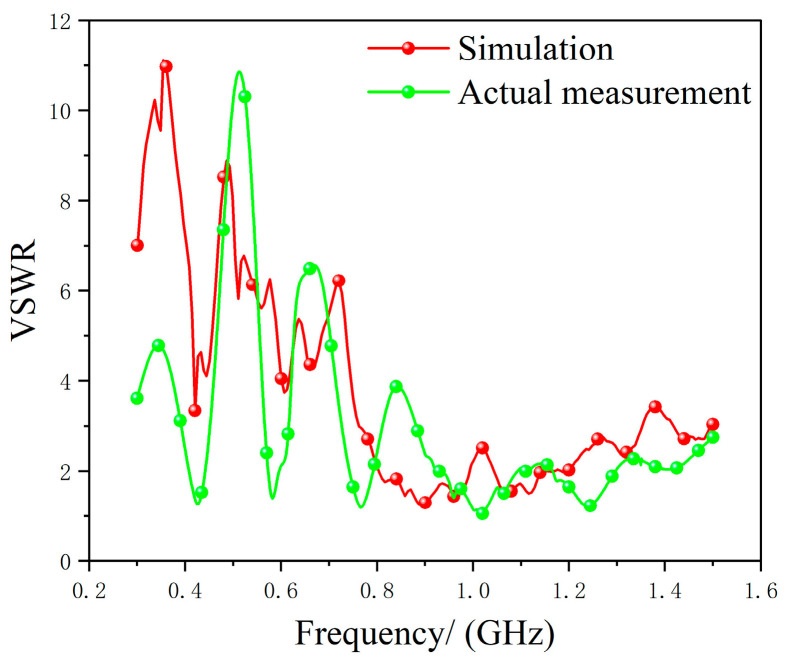
Comparison of Simulated and Measured VSWR.

Simulation and experimental error analysis were then conducted, comparing the results based on the standing wave ratio. The results are shown in Figure 16.

As shown in Figure 16, there are slight differences between the simulated and measured standing wave ratios, with the most significant discrepancy occurring in the low-frequency band of 0.3–0.4 GHz. This primarily stems from the difference between the ideal excitation used in simulation and the manually soldered feedline employed in the actual measurement.

**Figure 17:**
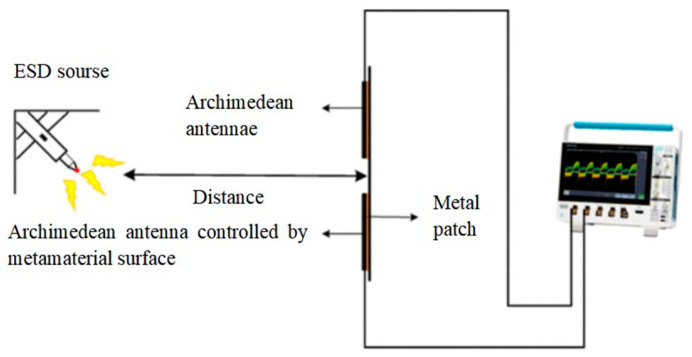
Electrostatic discharge detection schematic diagram.

**Figure 18:**
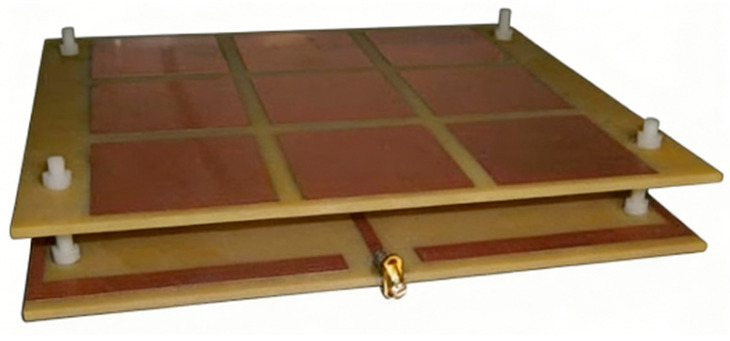
Resonant cavity monopole UHF antenna.

After completing the standing wave ratio test, the team constructed an electrostatic discharge (ESD) testing platform [22], using a resonant cavity monopole UHF antenna [23] as a reference antenna. ESD tests were conducted on the conventional Archimedean antenna, the metasurface-controlled Archimedean antenna, and the resonant cavity monopole UHF antenna. Schematic diagrams of the ESD test circuit and the resonant cavity monopole UHF antenna are shown in Figure 17 and Figure 18, respectively.

**Figure 19:**
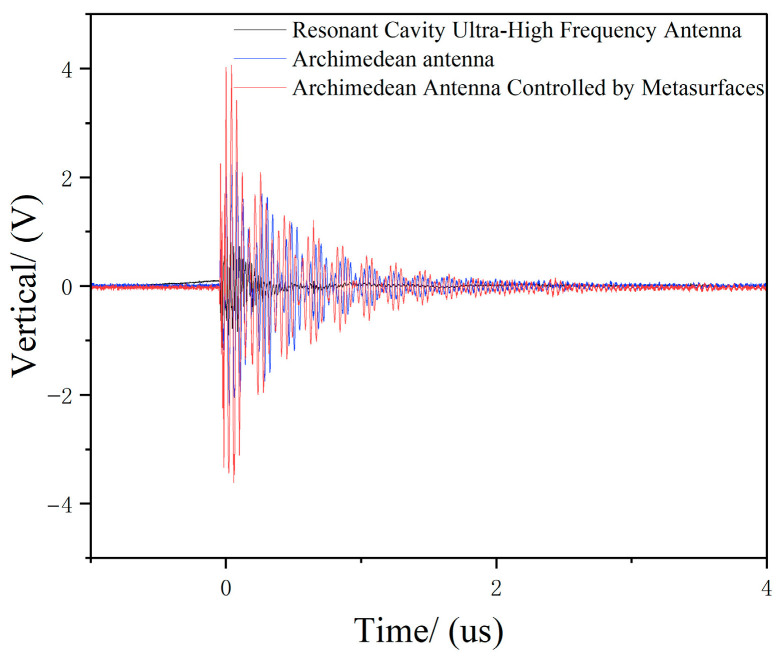
Waveforms of UHF signals detected by each antenna during electrostatic discharge.

We recorded the UHF signal amplitude detected by the Archimedean antenna, the Archimedean antenna controlled by a metasurface, and the resonant cavity monopole UHF antenna displayed on 10 oscilloscopes. The average values were used as statistical data for comparison. The UHF signal amplitude and waveform detected by each antenna during electrostatic discharge are shown in Table 3 and Figure 19, respectively.

As shown in Table 3 and Figure 17, during electrostatic discharge, the UHF signal amplitude detected by the metasurface-enhanced Archimedean antenna was 3881.8 mV, while the conventional Archimedean antenna was recorded as 2055.2 mV, and the resonant cavity monopole UHF antenna was recorded as 1017.2 mV. The super-surface-controlled Archimedean antenna demonstrated an approximately 88.9% increase in amplitude compared to the conventional Archimedean antenna, indicating that this super-surface significantly enhances the electrostatic monitoring performance of the Archimedean helical antenna. The signal amplitude of the metamaterial-enhanced Archimedean antenna on the oscilloscope was markedly higher than that of the conventional Archimedean antenna and the resonant cavity monopole UHF antenna.

**Figure 20:**
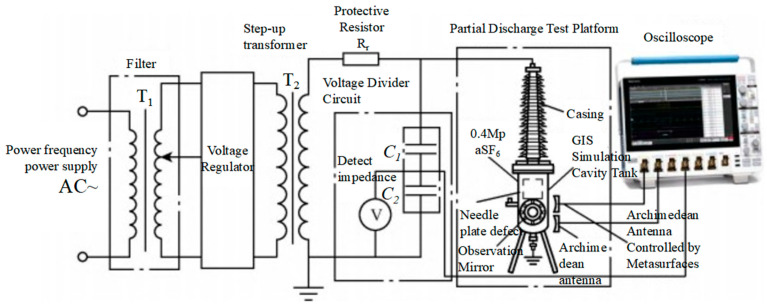
Schematic diagram of the GIS discharge testing circuit.

To further validate the enhancement effect of the metasurface on the UHF signal amplitude detection of the Archimedean antenna and its practical performance, the team constructed a GIS discharge platform [24] to conduct actual GIS defect discharge measurements on the Archimedean antenna, the metasurface-controlled Archimedean antenna, and the resonant cavity monopole UHF antenna. The GIS discharge detection circuit diagram is shown in Figure 20.

**Figure 21:**
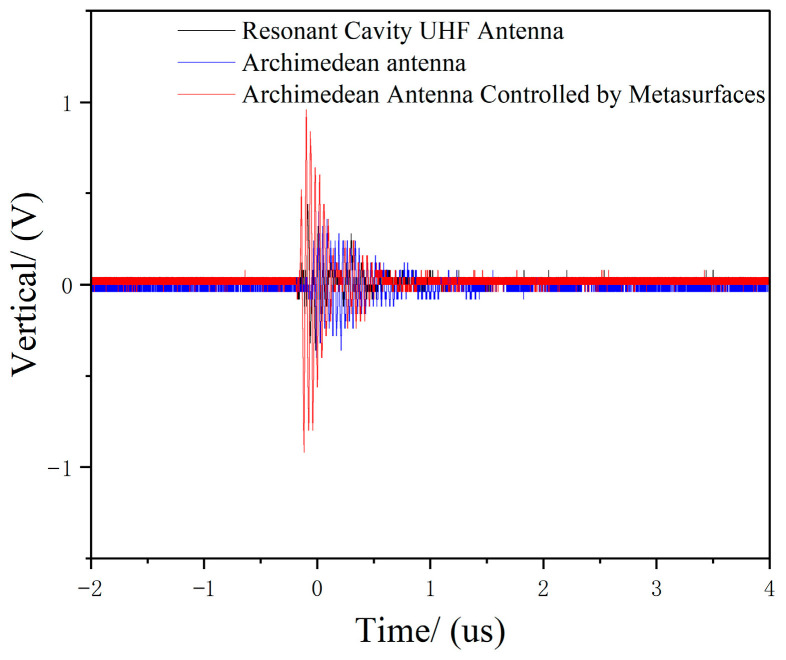
Detection of UHF signal waveforms by each antenna during GIS discharge.

Within the UHF signal amplitude range detectable by the oscilloscope, the power frequency supply voltage was steadily increased in 2 kV increments. The UHF signal amplitudes detected by the Archimedean antenna, the metamaterial-enhanced Archimedean antenna, and the resonant cavity monopole UHF antenna were recorded on the oscilloscope. The average of 10 data sets was taken as the UHF signal amplitude detected by each antenna during GIS discharge. The UHF signal amplitudes detected by each antenna during GIS discharge and the UHF signal waveforms detected by each antenna during GIS discharge at 6.6 kV are shown in Table 4 and Figure 21, respectively.

As shown in Table 4 and Figure 21, during GIS discharge, the pulse signal amplitudes of the Archimedean antenna, the metasurface-controlled antenna, and the resonant cavity monopole UHF antenna all exhibited an upward trend as the power frequency supply voltage gradually increased due to increased discharges. At 6.6 kV, the UHF signal amplitude detected by the metasurface-controlled Archimedean antenna reached 964.0 mV, compared to 404.0 mV for the conventional Archimedean antenna and 420.0 mV for the resonant cavity monopole UHF antenna. Compared to the Archimedean antenna, the super-surface-controlled Archimedean antenna exhibited a 138.6–150% increase in amplitude across five voltage levels, indicating a significant enhancement in UHF detection sensitivity following super-surface regulation.

## 4. Results

To address insufficient antenna gain in UHF applications at substations, this paper designed a high-sensitivity PD Archimedean antenna based on a rotating-unit reflective metasurface. Through simulation and experimental analysis, the following conclusions were drawn:

1. By incorporating a reflective phase-modulating metasurface, the Archimedean antenna achieved an average gain enhancement of 3.42 dB across the 0.3–1.5 GHz band, with a maximum gain enhancement of 15.61 dB.
2. PD measurements indicate that after super-surface phase modulation and reflection, the antenna’s detection amplitude of UHF signals under electrostatic discharge increased by approximately 88.9%. Under GIS discharge, the detection amplitude of UHF signals by the metasurface-modulated Archimedean antenna increased by approximately 138.6–150%.
3. This study provides valuable insights into highly sensitive PD regulation in power equipment. The next step involves refining the impedance matching method to optimize low-frequency return loss, while conducting performance testing at actual substations to improve practical implementation.

## Figures and Tables

**Table 1:** Dimensions of the Archimedean Antenna.

Parameter	Value/mm	Parameter	Value/mm
*r*_0_	12.5	*s*	1.5
*r_M_*	100.0	*w*	1.5
*r_M1_*	77.0	*d*	1.6

**Table 2:** Rotating Unit Reflection Metasurface Unit Dimensions.

Parameter	Value/mm	Parameter	Value/mm
*a*	20.0	*m*	3.6
*p*	0.8	*n*	6.4
*k*	0.8	*d_1_*	3.0

**Table 3:** Detection of UHF signal amplitudes by each antenna during electrostatic discharge.

Resonant Cavity Monopole UHF Antenna	Archimedean	Surface-Controlled Archimedean Antenna	Amplitude Increase
1017.2 mV	2055.2 mV	3881.8 mV	88.9%

**Table 4:** Detection of UHF signal amplitudes by each antenna during GIS discharge.

Voltage	Resonant Cavity Monopole UHF Antenna	Archimedean	Amplitude Increase After Metasurface Regulation	Amplitude Increase
6.6 kV	420.0 mV	404.0 mV	964.0 mV	138.6%
8.6 kV	420.0 mV	392.0 mV	980.0 mV	150.0%
10.6 kV	424.0 mV	396.0 mV	984.0 mV	148.5%
12.6 kV	424.0 mV	404.0 mV	988.0 mV	144.6%
14.6 kV	468.0 mV	448.0 mV	1076.0 mV	140.2%

## Data Availability

The original contributions presented in this study have been incorporated into the article, and the experimental data were obtained by the authors.
